# Soluble amyloid-β, effect on cerebral arteriolar regulation and vascular cells

**DOI:** 10.1186/1750-1326-5-15

**Published:** 2010-04-13

**Authors:** Hans H Dietrich, Chuanxi Xiang, Byung H Han, Gregory J Zipfel, David M Holtzman

**Affiliations:** 1 Department of Neurological Surgery, Hope Center for Neurological Disorders, and Alzheimer's Disease Research Center, Washington University School of Medicine, St Louis, MO, 63110, USA; 2 Department of Neurological Surgery, Washington University School of Medicine, St Louis, MO, 63110, USA; 3 Departments of Neurological Surgery, Neurology, and Hope Center for Neurological Disorders, Washington University School of Medicine, St Louis, MO, 63110, USA; 4 Department of Neurology, Hope Center for Neurological Disorders, and Alzheimer's Disease Research Center, Washington University School of Medicine, St Louis, MO, 63110, USA

## Abstract

**Background:**

Evidence indicates that soluble forms of amyloid-β (Aβ) are vasoactive, which may contribute to cerebrovascular dysfunction noted in patients with Alzheimer's Disease and cerebral amyloid angiopathy. The effects of soluble Aβ on penetrating cerebral arterioles - the vessels most responsible for controlling cerebrovascular resistance - have not been studied.

**Results:**

Freshly dissolved Aβ_1-40 _and Aβ_1-42_, but not the reverse peptide Aβ_40-1 _constricted isolated rat penetrating arterioles and diminished dilation to adenosine tri-phosphate (ATP). Aβ_1-42 _also enhanced ATP-induced vessel constriction. Aβ_1-40 _diminished arteriolar myogenic response, and an anti-Aβ antibody reduced Aβ_1-40 _induced arteriolar constriction. Prolonged Aβ exposure in vessels of Tg2576 mice resulted in a marked age-dependent effect on ATP-induced vascular responses. Vessels from 6 month old Tg2576 mice had reduced vascular responses whereas these were absent from 12 month old animals. Aβ_1-40 _and Aβ_1-42 _acutely increased production of reactive oxygen species (ROS) in cultured rat cerebro-microvascular cells. The radical scavenger MnTBAP attenuated this Aβ-induced oxidative stress and Aβ_1-40_-induced constriction in rat arterioles.

**Conclusions:**

Our results suggest that soluble Aβ_1-40 _and Aβ_1-42 _directly affect the vasomotor regulation of isolated rodent penetrating arterioles, and that ROS partially mediate these effects. Once insoluble Aβ deposits are present, arteriolar reactivity is greatly diminished.

## Background

Amyloid beta (Aβ) peptides are naturally occurring cleavage products of the amyloid precursor protein and produced via β- and γ-secretase resulting in soluble Aβ monomers [1]. Important species include peptides containing 40 and 42 amino acid residues (Aβ_1-40 _and Aβ_1-42_) [2]. Monomers of Aβ can aggregate resulting in deposits of fibrillar Aβ both as neuritic plaques and, within blood vessels, as cerebral amyloid angiopathy (CAA). Such deposits are hallmarks of Alzheimer's Disease (AD) [1]. Several lines of evidence suggest that not only Aβ aggregates but soluble Aβ species may also contribute to AD with its vasoactive properties. Cerebral hypoperfusion prior to the onset of AD has been observed [3]. In animal models of AD, some but not all studies suggest that soluble Aβ may decrease myogenic response, cerebral blood flow (CBF) and vasodilator responses [4-10]. This implies that soluble Aβ may have an effect on vascular regulation possibly affecting neuronal function [5], potentially contributing to ischemic brain damage [4]. To elucidate the vascular effects of soluble Aβ on the cerebral circulation, studies infusing soluble Aβ into rat [11] or superfusing mouse cortex [12] found that soluble Aβ acutely decreased CBF and response to vasodilators, it also increased vascular resistance and constrictor response. In *ex vivo *studies, soluble Aβ causes cerebral artery constriction, reduced dilation and/or increased constriction to endothelium-dependent dilators and vasoconstrictors, respectively [7,12-16]. The mechanism(s) by which soluble Aβ interferes with vascular function is not fully understood. But increased production of reactive oxygen species (ROS) has been described [15,17-19]. However, other mechanisms such as increased intracellular calcium activity [16] or decreased endothelial nitric oxide availability [20] have been reported. Because endothelium-dependent dilation rather than smooth muscle function [4,15] was impaired, studies concluded that soluble Aβ causes endothelial dysfunction resulting in the observed reduced vasomotor function [4,15,16].

To date, studies examining the functional effects of soluble Aβ on the cerebro-vasculature have been limited to cortical CBF measurements following topical application of soluble Aβ [12] and measurements of isolated cerebral arteries exposed to soluble Aβ [12,20]. No data regarding intracerebral micro-vessels exist. These vessels are exposed to both soluble Aβ as sites of Aβ clearance [21] and substantial CAA which forms in the vessel wall. Penetrating arterioles contribute significantly to the local regulation of CBF by controlling as much as 25% of total arterial resistance, exercising strong myogenic response [22] and differ in physiological response from proximal pial vessels and arteries [23]. For these reasons, it is important to examine the effects of Aβ on these critical microvessels and determine which mechanisms are involved. Finally, the effect of prolonged exposure to endogenous Aβ on cerebral arterioles has not been previously described.

Here we show strong effects of both soluble Aβ_1-40 _and Aβ_1-42 _on both vasoconstriction and vasodilation in penetrating arterioles, these effects are mediated in part by ROS. Further, prolonged exposure to high levels of Aβ in a mouse model with CAA was accentuated with very poor arteriolar function.

## Methods

### Isolation and cannulation of penetrating arterioles

All procedures were approved by the Washington University Advisory Committee for Animal Resources. Male Sprague-Dawley rats (350-450 g, Harlan, Indianapolis, IN) were anesthetized with pentobarbital sodium (65 mg/kg intraperitoneally) and sacrificed. Transgenic Tg2576 mice (gift of K. Hsaio) and their wild type litter mates on a B6/SJL background were bred in our animal facilities. The mice were anesthetized with Ketamine/Xylazine and sacrificed. The cerebral penetrating arterioles were excised from the distribution of the middle cerebral artery. Arterioles with a length of 500 to 1000 μm were transferred to an organ bath (2.5 ml volume) mounted on the stage of an inverted video microscope (Zeiss 100TV or Zeiss 200), and cannulated with glass micropipettes. No intraluminal flow was applied and the transmural pressure was set at 50 mm Hg (mice) or 60 mmHg (rats) and continuously monitored. We observed the internal diameter of the vessels using a computerized diameter tracking system (Diamtrak, T.O. Neild, Flinders University, Adelaide, Australia) with a spatial resolution of 0.5 μm/pixel and a data acquisition rate of 10 Hz. Rat arterioles averaged a maximum passive diameter of 64.9 ± 13.6 μm. Mouse arterioles averaged maximum passive diameters of 51.8 ± 7.7 μm for WT littermates and 52.7 ± 7.0 μm for APPsw mice.

The arterioles were superfused continuously with a physiological saline solution (37.5°C; pH 7.3) of the following composition (in mmol/L): 144 NaCl, 3 KCl, 2.5 CaCl_2_, 1.4 MgSO_4_, 2.0 pyruvate, 5.0 glucose, 0.02 ethylenediaminetetraacetic acid (EDTA), and 2.0 3-(N-morpholino) propanesulfonic acid (MOPS), 1.21 NaH_2_PO_4_. After equilibration, the vessels developed spontaneous tone and we confirmed their viability by changing the extraluminal pH from 7.3 to 6.8 and from 7.3 to 7.65. Vessels with poor tone (less than 20% decrease from the maximum diameter) or poor response to pH (less than 15% change in diameter after pH change) were excluded.

### Pharmacological studies

Stock solutions of commercially available amyloid β peptides were prepared in distilled water (one mmol/L), kept frozen until use and diluted in physiological buffer just prior to use. Using Western blot, we confirmed that the preparations contained predominantly monomeric peptides (data not shown). Similarly, adenosine tri-phosphate (ATP) stock was prepared in distilled water (10 mmol/L) and kept frozen until use. Mn(III) tetrakis (4-benzoic acid) porphyrin (MnTBAP), catalase and superoxide dismutase (SOD) were used to scavenge oxygen radicals. For dose response or agonist studies, the vessel where pretreated with respective Aβ concentrations for 20 minutes. Agonists such as ATP where applied in the presence of amyloid β. Purinergic P2X1 receptors were inhibited with pyridoxalphosphate-6-azophenyl-2',4'-disulphonic acid (PPADS, 3 μmol/L) [24].

Amyloid peptides were purchased from American Peptide Co. (Sunnyvale, CA); all other chemicals were obtained from Sigma (St. Louis, MO).

CAA coverage on TG2576 mouse cerebral arterioles was visualized according to our previously published method using Thioflavin-S (0.005% in MOPS buffer) to visualize CAA. Qualitatively, the vessels in this study were affected to the same extent by CAA as was shown in our previous publication [8].

### Cell Culture

Rat cerebral microvascular endothelial and smooth muscle cell lines (obtained from Dr. Diglio, Wayne State University, Michigan) [25,26] were cultured in antibiotic free DMEM with 10% FCS at 37°C at 95% CO_2 _and 5% air. Though cell cultures may have undergone changes compared to native cells, in preliminary experiments we confirmed that the endothelial cells tested positive for endothelial nitric oxide and the smooth muscle cells for f-actin thus retaining their respective main phenotype. Cell suspensions were plated into 96 well plates and grown to near confluency. On the day of experiment, the cells were washed with warmed (37°C) Medium Leibovitz, loaded with the oxygen radical sensitive dye MitoTracker Red CM-H_2_XRos (Invtirogen, diluted in Medium Leibovitz at 5 μmol/L) and incubated for 15 to 20 minutes. After incubation, the cells were washed with warmed Medium Leibovitz, freshly dissolved Aβ was added (one and two μmol/L) immediately before the measurement and the change in fluorescence measured for 30 minutes at 37°C with a plate reader (Synergy HTTR with KC4 software, Biotek Instruments, Ex = 475 ± 15 nm, Em = 645 ± 40 nm). Oxidation of MitoTracker Red CM-H_2_XRos by ROS increases the dye's fluorescence 100-fold rendering it very sensitive as a detector of ROS. In preliminary experiments we also tested 100 nM Aβ which had insignificant effect and was not used further.

### Statistics

All data are presented as mean ± SEM, with n representing the number of observations. Statistics were conducted on absolute vessel diameters. Differences were considered significant at p < 0.05 and determined by repeated-measures analysis of variance RANOVA with Student-Newman-Keuls test as post test or paired Student's t-test where appropriate. For the Aβ dose response studies, the data are presented as relative diameter change (% relative vessel Diameter = D_ATP_/D_Tone _*100) where D_Tone _is the baseline diameter of the vessel before the stimulation with ATP, and D_ATP _is the diameter of the vessel after the stimulation. For experiments with Aβ pre-incubation and mouse vessels the data are presented as percent maximal diameter and was calculated by the following formula: % maximum dilation = [(D_ATP _- D_tone_)/(D_max _- D_tone_)]* 100, where D_max _is the maximum diameter of the vessel at 60 mm Hg before the development of spontaneous tone [24,27]. This method corrects for changes in arteriolar to due to Aβ incubation. Changes in fluorescence intensity are presented as percent change from time zero time over 30 minutes of observation time.

## Results

### Extraluminal application of soluble Aβ_1-40_, Aβ_1-42 _the reverse peptide Aβ_40-1 _on arteriolar tone and myogenic response

**Figure 1:**
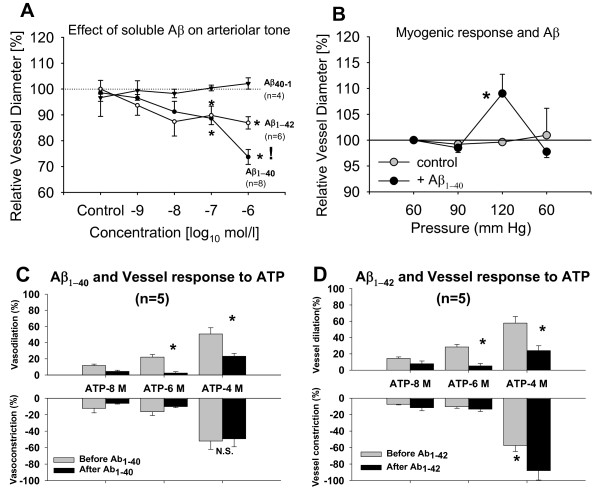
**Effect of freshly dissolved Aβ on rat penetrating arteriolar vessel tone, myogenic response and effect on ATP induced vasomotor responses**. Cerebral arterioles *ex vivo *were treated with increasing concentrations of Aβ_1-40_, Aβ_1-42 _or reverse peptide Aβ_40-1_ and the effect on arteriolar tone measured. **(A**)Aβ_1-42 _constricts the arterioles similarly to Aβ_1-40 _at 0.1 μmol/L while at 1 μmol/L Aβ_1-40 _has a greater effect. * and ! denote p < 0.05 from control (ANOVA). The reverse peptide Aβ_1-40 _had no effect. (**B**) Arterioles pretreated with Aβ_1-40 _(0.1 μmol/L) lose myogenic response at 120 mmHg intraluminal pressure. Returning to 60 mmHg baseline pressure reestablished control tone diameter. * denotes p < 0.05 from control (n = 3 vessels). Cerebral arterioles were treated with Aβ and vasomotor responses to extraluminal ATP were observed. (**C**) Aβ_1-40 _and (**D**) Aβ_1-42 _reduce vasodilation to ATP at 1 and 100 μmol/L concentrations. In addition Aβ_1-42 _also enhances constriction at 100 μmol/L ATP. * denotes p < 0.05 between before and after amyloid peptide (ANOVA).

Rat penetrating cerebral arterioles exposed to Aβ_1-40 _had a maximum diameter of 63.2 ± 4.1 um and developed a spontaneous tone diameter of 46.9 ± 3.3 um (n = 8). Freshly dissolved Aβ_1-40 _resulted in constriction of penetrating arterioles in a dose dependent manner which was significant at a concentration of 0.1 and 1 μmol/L resulting in a reduction of the tone diameter by 26.3 ± 2.9% (Figure 1A).

Vessel exposed to Aβ_1-42 _had a maximum passive diameter of 78.0 ± 8.7 μm and developed a spontaneous tone diameter of 56.8 ± 7.1 μm. Freshly dissolved Aβ_1-42 _constricted the penetrating arterioles significantly at 0.1 and 1μmol/L, an effect similar to that seen with Aβ_1-40 _(Figure 1A). However, at one μmol/L Aβ_1-40_, there was a 13% stronger constriction compared to Aβ_1-42 _(p < 0.05).

Vessels treated with the reverse peptide Aβ_40-1 _had a maximum diameter of 64.8 ± 7.2 μm and a spontaneous tone diameter of 46.6 ± 5.6 μm (n = 4). The reverse peptide had no effect on the tone diameter (Figure 1A).

Myogenic response describes the ability of arterial blood vessels to maintain a constant vessel tone over a range of intraluminal pressures, a mechanism important for cerebral autoregulation. The arterioles used had a maximum diameter of 76.5 ± 13.5 μm and a tone diameter of 60.7 ± 10.7 μm before and of 51.5 ± 9.3 μm after Aβ_1-40 _(0.1 μmol/L). Under control conditions, vessels maintained their diameter as intraluminal pressure increased from 60 mmHg to 90 mmHg and 120 mmHg. After incubation with freshly dissolved Aβ_1-40 _(0.1 μmol/L) the vessels' ability to maintain myogenic responses was eliminated at 120 mmHg intraluminal pressure but not at 90 mmHg. The vessels returned to base line diameters at 60 mmHg indicating that the pressure increase did not permanently damage the vessels (Figure 1B).

### Arteriolar response to adenosine tri-phosphate (ATP) and soluble Aβ_1-40 _and Aβ_1-42_

Extraluminal ATP causes a biphasic vessel response with a transient constriction caused by smooth muscle P_2X1_-receptors and a subsequent endothelium dependent dilation via P_2Y_-receptors [24] thus allowing studies of both smooth muscle- and endothelium-dependent responses. We confirmed that ATP causes a transient constriction via P_2X1 _stimulation in mouse cerebral arterioles. PPADS (3 μmol/L) significantly inhibited constriction to ATP (100 μmol/L) from -16.6 ± 1.2% to -1.3 ± 0.7% (n = 3) while dilation to ATP was unchanged (data not shown). Arterioles treated with Aβ_1-40 _had a passive maximum diameter of 58.6 ± 3.7 μm and spontaneously constricted to 41.2 ± 3.6 μm (n = 5). Vessels treated with Aβ_1-42 _had a maximum diameter of 69.5 ± 5.4 and a tone diameter of 48.2 ± 4.1 μm. Both freshly dissolved Aβ_1-40 _and Aβ_1-42 _(one μmol/L) significantly decreased dilation to ATP indicating that endothelium dependent dilation was reduced (Figure 1C and 1D). While the constrictory response to ATP was unchanged in the presence of Aβ_1-40_, we observed an enhanced smooth muscle-dependent constriction with Aβ_1-42 _with 100 μmol/L concentrations of ATP (Figure 1D). Repeated application of ATP alone (100 μmol/L) did not change the vessel response (relative first constriction of -66.6 ± 7.1% and dilation of 46.2 ± 5.4% versus repeated constriction of -53.2 ± 6.4% and 39.2 ± 7.2% dilation, n = 4 vessel, repeated measures ANOVA).

### Soluble Aβ and ROS

**Figure 2:**
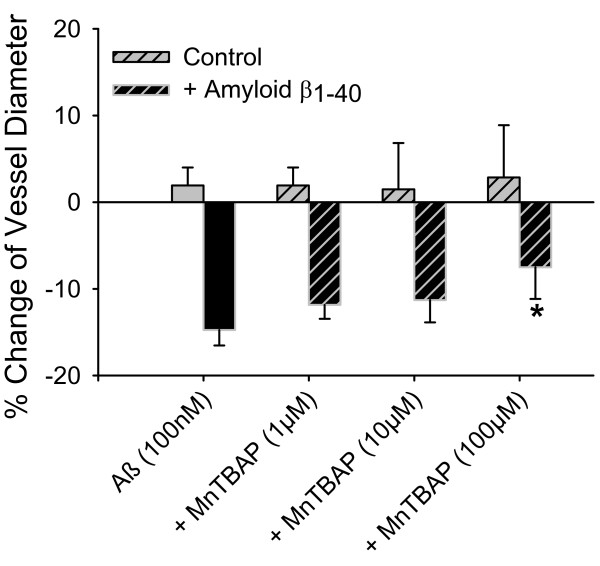
**Attenuation of Aβ_1-40_-induced vessel constriction by ROS scavenging**. Cerebral arterioles were incubated with 100 nmol/L Aβ_1-40 _and/or the ROS scavenger MnTBAP. MnTBAP reduced Aβ_1-40_-induced vessel constriction at 100 μmol/L indicating that the Aβ effect is partially due to reactive oxygen species. * denotes p < 0.05 from Aβ alone (n = 3). MnTBAP itself had no effect on the vessel diameter (n = 5).

Soluble Aβ may cause ROS production in cerebral vessels. We applied the reactive oxygen scavenger MnTBAP (1 to 100 μmol/L) and found that in the presence of Aβ_1-40 _(1 μmol/L) MnTBAP partially restored vessel tone and diameter at the highest MnTBAP concentration (Figure 2). MnTBAP itself had no effect on the vessel diameter.

### Microvascular endothelial and smooth muscle cells, Aβ_1-40 _and Aβ_1-42 _and ROS

**Figure 3:**
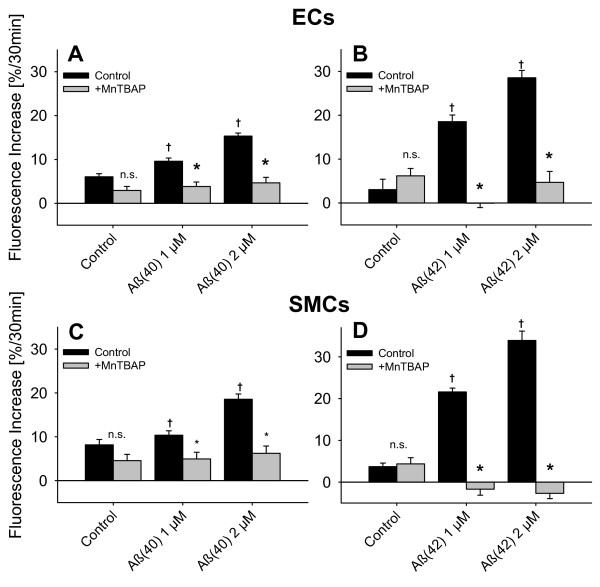
**Dose dependent increase of oxygen radical production induced by soluble Aβ in cultured rat cerebral microvascular cells**. To quantify Aβ-induced ROS production in vascular cells, rat cerebral microvascular endothelial and smooth muscle cells were incubated with soluble Aβ (1 and 2 μmol/L) and ROS production was measure using the ROS sensitive dye MitoTracker Red CM-H_2_XRos (5 μmol/L). (**A**) Aβ_1-40 _and (**B**) Aβ_1-42 _increase dose-dependently ROS in endothelial ECs) and (**C **and **D**) smooth muscle cells (SMCs). * denotes p < 0.05 from control. In SMCs Aβ_1-42 _has a stronger effect compared to Aβ_1-40 _as indicated by † = p < 0.05. N ≥ four replicates with four cultures per replicate (n ≥ 16).   n.s. denotes not significant.

**Figure 4:**
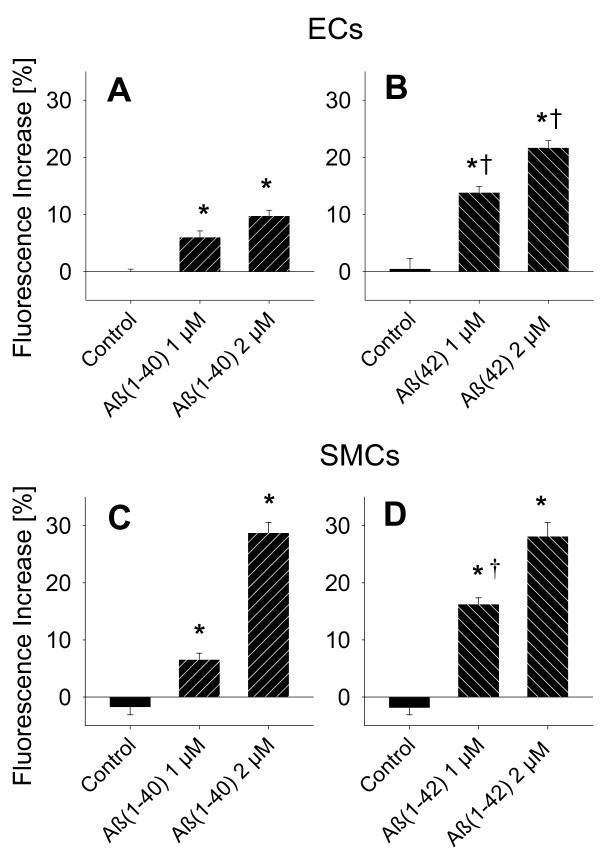
**Scavenging of oxygen radical production in rat cerebral microvascular endothelial and smooth muscle cells**. ROS production was measured in rat cerebral microvascular endothelial and smooth muscle cells using the ROS sensitive dye MitoTracker Red CM-H_2_XRos (5 μmol/L). MnTBAP (100 μmol/L) inhibits the increase in (**A**) Aβ_1-40_- and (**B**) Aβ_1-42_-induced oxygen radical production both in cultured rat cerebral microvascular endothelial cells and in smooth muscle cells (**C **and **D**). † denotes p > 0.05 from Control; * denotes p > 0.05 MnTBAP versus respective Control (ANOVA). N > three replicates with three cultures per replicate (n ≥ 9).

**Figure 5:**
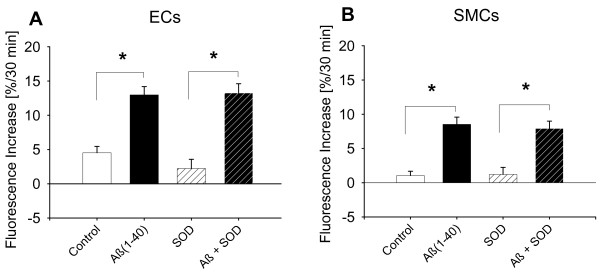
**Effect of superoxide dismutase (SOD) on Aβ-induced ROS increase**. ROS production was measured in rat cerebral microvascular endothelial and smooth muscle cells using the ROS sensitive dye MitoTracker Red CM-H_2_XRos (5 μmol/L). SOD was unable to attenuate Aβ_1-40_-induced reactive oxygen radical increase in either (**A**) endothelial (ECs) or (B) smooth muscle cells (SMCs). * denotes p > 0.05 from Control. N > three replicates with three cultures per replicate (n ≥ 9).

To study a possible contribution of reactive oxygen species in the observed vessel dysfunction, we loaded cultured rat cerebral microvascular endothelial or smooth muscle cells with the ROS sensitive dye MitoTracker Red CM-H_2_XRos. We found that after 30 minutes of incubation, both freshly dissolved Aβ_1-40 _and Aβ_1-42 _significantly and dose dependently increased ROS production in the two cell types with Aβ_1-42 _having a greater effect in endothelial cells compared Aβ_1-40 _(Figure 3A and 3B). In smooth muscle cells, Aβ_1-42 _was more effective at 1 μmol/L than Aβ_1-40 _in producing ROS (Figure 3C and 3D). Aβ (100 nM) induced production of ROS was inhibited by the ROS scavenger MnTBAP (Figure 4), but not by SOD (60 U/ml, Figure 5) or Catalase (40 U/ml, data not shown). These data correspond with our *ex vivo *observation that MnTBAP reduces the effect of Aβ indicating that ROS are involved in the observed vascular dysfunction. However, it is possible that cultured cells may not correctly reflect in vivo situations.

### Arteriolar vasomotor responses and chronic exposure to soluble Aβ

**Figure 6:**
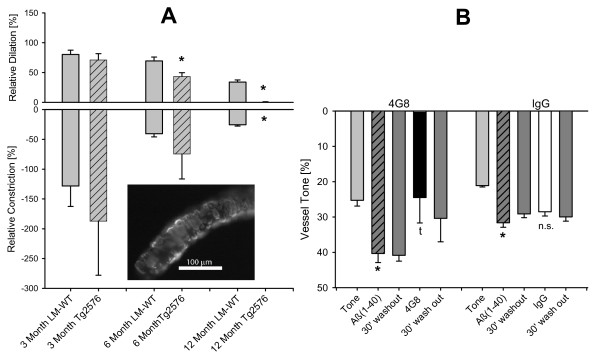
**Vasomotor responses to ATP in penetrating arterioles of 3, 6 and 12 months old Tg2576 and WT litter mates and attenuation of Aβ_1-40 _induced vessel constriction by 4G8 antibody**. Vasomotor responses to extraluminal ATP were measured in cerebral arterioles of control littermate and TG2576 mice *ex vivo*. (**A**) At three months of age, the vessels show strong responsiveness to extraluminal ATP both in Tg2576 and WT-LM. At six months, dilation to ATP is reduced in Tg2576 mice. At 12 months, vasomotor responses in Tg2576 vessels are greatly reduced and the vessels show smooth muscle cells enveloped by amyloid. Insert: Thioflavin-S staining of an arteriole (44 μm diameter) from a Tg2576 mouse used in the experiments. * denotes p > 0.05 from WT at same age. (B) Incubation with Aβ_1-40 _caused a significant arteriolar constriction which lasted at least for 30 minutes after washout. The soluble Aβ-specific antibody 4G8 restored control vessel tone while the control IgG did not. * = p < 0.05 from tone diameter for both control IgG and 4G8 treated vessels; t = p < 0.05 from Aβ_1-40_-induced constriction; n.s = not significant from Aβ_1-40_-induced constriction.

We compared vasomotor responses to ATP *ex vivo *in penetrating arterioles from Tg2576 mice of 3, 6 and 12 months of age compared to age matched wild type littermates. Tg2576 mice develop Aβ deposition in the brain and arterioles beginning at 9 months of age [28,29]. Wild type vessels had maximum diameter of 53.5 ± 6.5 μm, 61.8 ± 3.6 μm and 54.8 ± 5.3 μm at 3, 6 and 12 months of age, respectively. The tone development was 16.5 ± 6.5 ± 1.2% (n = 24), 13.3 ± 2.9% (n = 4) and 9.8 ± 6.2% (n = 4). Vessels from Tg2576 mice had a maximum diameter of 54.0 ± 3.5 μm, 55.8 ± 2.8 μm and 54.6 ± 2.6 μm at 3, 6 and 12 months of age, respectively. The tone development was 6.8 ± 0.5% (n = 3), 26.5 ± 8.0% (n = 4) and 1.8 ± 1.4% (n = 6). We found no difference in the responses to ATP 3 at months (Figure 6A). At six months of age dilatory response to ATP was reduced in the Tg2576 vessels compared to wild type vessels. At 12 months of age, both dilatory and constrictory vasomotor responses to ATP were virtually absent in Tg2576 vessels while the wild type vessels still showed a constriction to ATP followed by a small dilation (Figure 6A). We measured amyloid coverage in three vessels which was 67.9 ± 4.0%. To test if the vessels from the 12 month old Tg2576 mice can respond to other vasoactive stimuli, we applied alkaline buffer of pH 7.65 and observed a constriction of 10.9 ± 5.2%, indicating that the arterioles still have some capacity to respond other stimuli.

### Extraluminal application of anti-Aβ antibody 4G8

Passive immunization with certain anti Aβ antibodies has shown that such treatment can rapidly improve cognitive function in animal models of AD [30]. We tested the hypothesis that antibody treatment can interfere with the effect of soluble Aβ on cerebral arterioles. Arterioles were first treated with freshly dissolved Aβ_1-40_, the Aβ washed out, treated with the anti-Aβ antibody 4G8 antibody (1:200 dilution) and finally the 4G8 washed out. Each treatment applied for 30 minutes and the diameter measured at the end of each treatment. Freshly dissolved Aβ_1-40 _significantly constricted the arterioles and this constriction remained even after 30 minutes of washout. Incubation with 4G8 antibody with the vessels, 30 minutes after Aβ_1-40 _washout, restored vessel tone (Figure 6B). For control experiments we replaced 4G8 antibody with non-specific IgG antibody. In this control experiment non-specific IgG did not restore vessel tone showing that Aβ_1-40 _has a prolonged vasoconstrictive effect even after prolonged washout (Figure 6B). This indicates that Aβ_1-40 _may interact with the vessel even after washout and that antibody to soluble Aβ may interfere with this interaction.

## Discussion

Our study has several notable findings, including the following: 1) freshly dissolved Aβ_1-40 _and Aβ_1-42_, but not the reverse peptide Aβ_40-1_, acutely increase vessel tone in rat cerebral penetrating arterioles, with Aβ_1-42 _being equally potent to Aβ_1-40 _except at the highest concentration; 2) Aβ_1-40 _and Aβ_1-42 _both decrease endothelium-dependent dilation to ATP, with Aβ_1-42 _also enhancing smooth muscle dependent constriction; 3) Aβ_1-40 _decreases arteriolar myogenic response; 4) MnTBAP attenuates Aβ_1-40_-induced increase in vessel tone increase suggesting that reactive oxygen species have a contributing role in Aβ-induced enhancement in vessel tone; 5) Aβ_1-40 _and Aβ_1-42 _acutely increased ROS production in cultured rat cerebral microvascular endothelial and smooth muscle cells in a dose dependent fashion - a response that was inhibited by the cell permeable ROS scavenger MnTBAP but not SOD or catalase; 6) Incubation of penetrating arterioles with Aβ_1-40 _for 30 minutes resulted in prolonged increased tone which was not restored by Aβ washout but was restored by the anti-Aβ antibody, 4G8; and 7) exposure to soluble as well as deposited Aβ decreased vascular reactivity over time in arterioles from Tg2576 mice, an animal model of AD. These results confirm many previous reports but also extend our understanding of the vascular effects of soluble Aβ, especially in regards to the pathological effects of Aβ_1-42 _on vessels and vascular cells. These results indicate a direct effect of the studied Aβ species on cerebral penetrating arterioles with potentially important differences in their activity such as enhanced arteriolar vasoconstriction to ATP or increased ROS production by Aβ_1-42 _but increased vessel constriction by Aβ_1-40_.

The penetrating cerebral arteriole is an important regulator of CBF representing about 25% of the total cerebral arterial regulatory capacity. Further, it is the part of the cerebral circulation most profoundly involved in the trafficking of Aβ from the brain [21] and continuously exposed to Aβ. Though the concentration of Aβ in the CSF is in the nanomolar range [31] the concentration in the periarteriolar space is not known and could be higher especially when transport across the arteriole is compromised and Aβ accumulates. Such accumulation would eventually lead to CAA [32]. So far no study has directly addresses the effect of soluble Aβ in this essential microvessel.

### Soluble Aβ

In CAA, amyloid deposition consists predominantly of Aβ_1-40 _rather than Aβ_1-42 _species [33]; however, in most cases of CAA, Aβ_1-42 _may initially be necessary to induce amyloid deposition [34]. Thus initial studies related to the effect of freshly dissolved Aβ on cerebral vessels that concentrated on Aβ_1-40 _[15]. Niwa et al. directly compared the effects of soluble Aβ_1-40 _versus Aβ_1-42 _in mouse cerebral circulation and found that Aβ_1-40 _but not Aβ_1-42 _recapitulated the observed vascular dysfunction in a mouse model of AD with a high Aβ_1-40 _to Aβ_1-42 _ratio [5,6,8]. Similarly, in rat aorta, Aβ_1-42 _was less effective than Aβ_1-40 _[35]. Our data indicate that at concentrations up to 100 nmol/L both Aβ species have a similar effect on rat cerebral arteriolar tone (though at concentrations higher than 100 nmol/L Aβ_1-40 _produced greater arteriolar tone). Previous studies found that Aβ_1-40 _reduced endothelium dilation to variety of agonists as well as physiological whisker stimulation [15,19] while Aβ_1-42 _and the reverse peptide Aβ_40-1 _did not [5,6,12]. Adenosine tri-phosphate (ATP) is an important cerebral vasoactive agonist which can be released from numerous sources including cerebral purinergic nerves, astrocytes and red blood cells [36]. Extraluminally applied ATP causes a biphasic diameter response in cerebral arterioles with an initial transient constriction due to smooth muscle cells purinergic P_2X1_-receptors [24,36] which quickly desensitize [37] followed by an endothelium-dependent dilation via P_2Y_-receptors [24,36]. The effect of Aβ species on ATP-induced vasomotor responses has not been reported previously. We found that similar to other endothelium-dependent agonists, Aβ_1-40 _and Aβ_1-42 _reduce dilation to ATP. Agonist-induced constriction with endothelin or serotonin is increased after Aβ_1-40 _[15]; however, this enhanced constriction was attributed to decreased endothelial function rather than an effect on smooth muscle cells [5,6,15]. In mice intraluminal Aβ_1-40 _caused an endothelin-dependent decrease in CBF which was attenuated by inhibiting the receptor for advanced glycation end products (RAGE) [38]. In our study we found that Aβ_1-42 _but not Aβ_1-40 _enhances constriction to ATP. This constrictive response to ATP is mediated via smooth muscle cells (endothelial denudation does not affect ATP-induced vasoconstriction [36]) and is likely due to desensitization of the P_2X1_-receptor responsible for constriction by limiting the time of its activity [36]. The enhanced constriction observed with Aβ_1-42 _likely means that it has a direct effect on smooth muscle cell function either by Aβ_1-42 _prolonging the open probability of the P_2X1_-receptor or by enhancing the sensitivity of the smooth muscle contractile apparatus. Freshly dissolved Aβ_1-40 _also decreases arteriolar myogenic response. That increased Aβ may decrease myogenic response was shown in a mouse model of AD [6].

Passive immunization with anti-Aβ antibodies is being evaluated as a possible treatment for AD [39]. Dodart et al. showed that passive immunization could rapidly (within 24 to 72 hours) improve cognitive function in an animal model of AD [30]. We therefore hypothesized that an antibody against Aβ may rapidly ameliorate Aβ-induced increase in arteriolar tone. We found that a 30 minute incubation with Aβ_1-40 _resulted in a persistent (> 90 minutes) increase in arteriolar tone despite repeated washouts. This indicates that, once in contact with the vessel, Aβ_1-40 _retains its vasoactive effect even in the absence of extravascular Aβ for some time. However, after application of the anti-Aβ antibody 4G8, the arteriolar tone was restored while non-specific IgG had no effect. This observation is consistent with the notion that Aβ_1-40 _resides on the cell surface in such a way that only high affinity binding agents can remove or neutralize. Alternatively the Aβ signaling mechanism may be slow to come on and slow to turn off. Further studies are needed to resolve the mechanism of soluble Aβ-induced arteriolar dysfunction. Taken together we present for the first time evidence that soluble Aβ_1-40 _as well as Aβ_1-42 _directly impair cerebral penetrating arteriolar function and that Aβ_1-42 _may be considered a vasoactive Aβ species in this preparation.

### ROS

The mechanism behind cerebral vascular dysfunction due to soluble Aβ is not completely understood. There is evidence that ROS are involved [15,17-19]. Since peroxynitrite did not duplicate soluble Aβ-induced vascular dysfunction in rat aorta [17], superoxide anion produced by NADPH oxidase may contribute to the observed vascular dysfunction [17,18]. Other studies suggest that soluble Aβ causes activation of COX-2 and other inflammatory responses resulting in the observed vascular dysfunction [13,40]. Soluble Aβ may trigger intracellular calcium mobilization which may activate calcium sensitive PKCs resulting in deactivation of eNOS [16].

We applied the cell permeant oxygen radical scavenger MnTBAP to cerebral arterioles *ex vivo *and found a partial restoration of arteriolar tone, indicating that oxygen radicals contribute to the increased tone development by Aβ. To further elucidate the acute effect of freshly dissolved Aβ on vascular cells, we measured ROS production in rat cerebro-microvascular endothelial and smooth muscle cells. Our results show, that freshly dissolved Aβ_1-40 _and Aβ_1-42 _dose-dependently increase ROS production in both endothelial and smooth muscle cells with Aβ_1-42 _having a greater ability to induce oxygen radical formation. This indicates that both Aβ species may acutely interfere with either cell type and their function in vascular regulation. Studies show that extraluminal Aβ_1-40 _damages cerebrovascular endothelium and impairs function within 30 minutes of incubation [15,40] indicating that Aβ quickly reacts with vascular cells. This suggests that Aβ may reach the abluminal endothelium to exert a direct endothelial effect similar to other small peptides such as bradykinin. While we and others found that Aβ directly affects endothelial cells in culture, experiments with extraluminal Aβ application are not consistent as to whether extraluminal Aβ directly affects the endothelium or if the Aβ effect is limited to the vascular smooth muscle from which a noxious mediator may diffuse to the endothelium. Catalase and superoxide dismutase (SOD) did not affect the Aβ-induced ROS production. MnTBAP is a cell permeable SOD-mimetic, as such MnTBAP scavenges O_2_^- ^at the source, presumably the mitochondria. SOD is not cell permeant and O_2_^- ^does not diffuse well out of the cell due to its polarity. As such SOD is expected to have less or no effect of scavenging intracellular ROS. Catalase metabolizes H_2_O_2 _to H_2_O and O_2_^-^. H_2_O_2 _is cell permeant and could be metabolized by extracellular catalase. However, since catalase did not change the ROS signal, H_2_O_2 _may not be involved in the observed ROS production. No study on the acute effect of Aβ-induced ROS on cerebral microvascular endothelial cells *in vitro *exists though cytotoxicity following a 24 hour exposure of cells incubated with Aβ_1-40_, was reduced by MnTBAP in RBE4 cells indicating that ROS were produced [17]. A consequence of oxidative stress is increased lipid peroxidation, measured e.g. as thiobarbituric acid reactive substances (TBARS), which was increased in cultured astrocytes after 24 hour incubation with Aβ_1-40 _[41]. While we were able to detect an increase in ROS with MitoTracker Red CM-H_2_XRos fluorescence, in preliminary experiments we could not detect an increase in lipid peroxidation with thirty minutes of Aβ incubation. It is likely that this incubation time is not long enough to result in a lipid peroxidation detectable by the TBARS method and more sensitive approaches such a mass spectroscopy have to be used.

Though no observations for acute effects exist, differences in the effect of Aβ species on endothelial cells were found in several studies. Folin et al. found that Aβ_1-40 _was less toxic to neuro-endothelial cells than Aβ_1-42 _[42] while the opposite was found for human cerebrovascular endothelial cells [43]. In other vascular endothelial cells it was found that Aβ_1-40 _reduces production of nitric oxide [16,20]. The effect of the vasculotropic Dutch (E693Q)/Iowa (D694N) mutant human Aβ_1-40_D was studied in human cerebro-vascular smooth muscle cells. The study found that Aβ_1-40_D did not increase ROS production [44]. Other mechanisms such as increased intracellular calcium activity [16] or decreased endothelial nitric oxide availability [20] have been reported which could also contribute to the observed vascular dysfunction.

### Chronic exposure to soluble Aβ

To test if chronic exposure to increased Aβ levels affects arteriolar vasomotor function over time, we compared arteriolar diameter responses of vessels from Tg2576 mice, a commonly used AD mouse model having elevated levels of soluble Aβ throughout life and age-dependent development of CAA [28], with age matched littermate controls. We found no difference in vessel responses to ATP at three months of age, but at six months of age, dilation to ATP was reduced in Tg2576 mice. There was also a trend towards increased tone and increased ATP-induced vasoconstriction, but this did not reach statistical significance. In 12 month old Tg2576 mice, the vasomotor response to ATP was essentially absent. Stimulation with alkaline pH still resulted in some constriction indicating that the vessels were responsive to some physiological stimulus. At this age, the vessels evaluated had CAA coverage of approximately 67%. Amyloid can also be sequestered in the basement membrane [45,46] which may be more likely to occur with basement membrane thickening that occurs with age. This will affect vessel functionality. Further accumulation of soluble Aβ could lead to formation of Aβ oligomers which in some studies are shown to be more effective/toxic than monomeric soluble Aβ [47]. The possible effect of Aβ oligomers on cerebral arterioles will be important to assess in future experiments. The lack of vasodilation in these vessels corresponds well with *in vivo *observations in pial arterioles where a CAA coverage of 50% or more eliminated dilatory response to hypercapnia [8]. The same study also found that in 12 month old TG2576 vessels, the smooth muscle content was significantly reduced. Thus increased CAA coverage and decreased smooth muscle content could, in part, explain the weak constrictory response observed at this age in our *ex vivo *studies.

Taken together these findings indicate that on the arteriolar level, chronic exposure to Aβ may lead to alterations in vasomotor function as early as six months of age when no amyloid deposits are present [5,8]. Though vessel function decreases with age, it deteriorates severely in Tg2576 animals. Relative CBF was found to be decreased in 13 month old Tg2576 mice [7]. CBF responses to a variety of stimuli in Tg2576 mice of 8 months (pre-CAA) was not different from age matched controls though they were depressed in 19 month old animals [9,10]. While our and the above studies did not observe vascular anomalies in Tg2576 mice younger than 6 months of age, Niwa et al. found decreased CBF responses in young (two to three months) Tg2576 mice [6]. It should be noted, however, that our data at 3 months of age did show a trend for decreased dilation and increased vasoconstriction in Tg2576 mice indicating that increased Aβ may have begun to effect cerebral arteriole function early in life.

## Conclusions

Utilizing isolated rat cerebral arterioles *ex vivo*, we found that both Aβ_1-40 _and Aβ_1-42 _cause vessel constriction and reduced dilation to ATP, with Aβ_1-42 _also enhancing arteriolar constriction response. Together with the observed loss of myogenic response, increased soluble Aβ levels may lead to pronounced vessel dysfunction *in vivo*. Both Aβ_1-40 _and Aβ_1-42 _increased oxygen radical production in cerebro-microvascular endothelial and smooth muscle cells which was inhibited by MnTBAP, indicating that oxygen radicals, in part, contribute to Aβ-induced penetrating arteriole dysfunction. The effects of Aβ-incubation on cerebral arterioles persists after washout but can be alleviated with an anti-Aβ antibody, suggesting that Aβ may exert its effects while bound to the cell surface. Chronic exposure to elevated Aβ results in cerebral microvascular dysfunction before CAA at six months of age which worsened with age resulting in minimal responsiveness at 12 months with CAA present. Overall the results suggest that soluble and fibrillar Aβ is influencing arteriolar function which may have an important effect on brain dysfunction in AD and CAA and may contribute to ischemia.

## Competing interests

The authors declare that they have no competing interests.

## Authors' contributions

HHD contributed to the general design of the *ex vivo *and *in vitro *experiments, performed *ex vivo *experiments and supervised the *in vitro *studies, and contributed to the statistical data analysis and the writing of the manuscript. CX performed additional *ex vivo *experiments and contributed to the statistical analysis. BHH performed the CAA coverage analysis. GJZ contributed to the design, analysis and interpretation of the studies, writing and review of the manuscript. DMH contributed to the overall experimental design, data interpretation and critical manuscript review. All authors have read and approved the final manuscript.
